# Roles of hCG in Advancing Follicular Growth to Ovulation after Concurrent Injections of PGF_2*α*_ and GnRH in Postpubertal Holstein Heifers Bearing a CL

**DOI:** 10.4061/2010/394236

**Published:** 2010-12-01

**Authors:** Ricky Johnson, William A. Bennett, Evelin J. Cuadra, Victor Njiti, Yoonsung Jung, Melissa Mason

**Affiliations:** ^1^Department of Agriculture, Alcorn State University, 1000 ASU Drive, Box 750, Alcorn State, MS 39096, USA; ^2^Department of Obstetrics and Gynecology, University of Mississippi Medical Center, 2500 North State Street, Jackson, MS 39216, USA; ^3^School of Research, Alcorn State University, 1000 ASU Drive no. 330, Alcorn State, MS 39096, USA

## Abstract

A study was conducted to test the hypothesis that injecting Gonadotropin-releasing hormone (GnRH) concurrently with Prostaglandin F2 alpha (PGF_2*α*_) followed by an injection of human Chorionic Gonadotropin (hCG), would advance follicular growth to ovulation in Holstein heifers bearing a corpus luteum (CL). After manual examination of the CL, group 1 (PGF; *n* = 12) received an injection of PGF_2*α*_ (25 mg, im). Group 2 (PGF + GnRH; *n* = 13) received an injection of GnRH (100 *μ*g, im) immediately after an injection of PGF_2*α*_. Group 3 (PGF + GnRH + hCG; *n* = 12) received concurrent injections of PGF_2*α*_ and GnRH followed with hCG (1500 IU, im) two days later. Follicular size and day of ovulation were monitored by daily ultrasonographic examination from days 1 to 10. Blood was collected on days-7, 0 (PGF_2*α*_ administration), 2, and 7. Progesterone was not different (*P* > .05) on days-7, 0, and 2 between the experimental groups. However, it was higher (*P* < .005) in the PGF + GnRH + hCG group on day 7 compared to PGF + GnRH heifers, but not significantly higher than the PGF. Additionally, heifers in the PGF + GnRH + hCG group ovulated earlier (*P* < .05) than heifers in the PGF + GnRH and the PGF group. This data indicates that hCG advances follicular growth to ovulation in spite of high levels of progesterone when injected 48 h after concurrent treatments of GnRH and PGF_2*α*_ on heifers bearing a CL.

## 1. Introduction

Over the past 25 years, the use of Gonadotropin-releasing hormone (GnRH) and Prostaglandin F2 alpha (PGF_2*α*_) has experienced a remarkable increase in research studies intended to manipulate ovarian cell response with the objective to improve cattle fertility. Even though significant progress has been made in this line of research, timing in the administration of GnRH in reference to PGF_2*α*_ and combination of these two hormones with others, are some of the topics of research at present. Thus, studies have been conducted in an attempt to improve pregnancy rates in CoSynch protocols by extending or reducing the intervals between GnRH injections and the PGF_2*α*_. Research trials have demonstrated the effectiveness of these protocols in improving pregnancy rates when PGF_2*α*_ is injected 7 days after administration of the first GnRH [1, 2]. Additionally, a study conducted by a group of investigators [3] showed that extending the interval between the first GnRH and the PGF_2*α*_ does not reduce the variability in response to synchronization of ovulation in heifers. However, others observed that extending the administration of the second GnRH to 48 h after the PGF_2*α*_ improved pregnancy rate [4]. On the other hand, drastically decreasing the interval between the first GnRH and PGF_2*α*_ may affect fertility. This is supported by findings indicating that the probability of pregnancy decreases substantially in dairy cows experiencing an incomplete corpus luteum (CL) regression [5]. The detrimental factor is the relatively high levels of progesterone secreted by luteal tissue as a consequence of an incomplete luteolysis [6] which in turn, reduces the ability of endogenous estradiol to induce a preovulatory surge of luteinizing hormone (LH) and ovulation [7]. High levels of progesterone are observed after concurrent administration of these two hormones due to either an incomplete regression of the CL [6] or formation of new luteal tissue in dairy heifers [8, 9]. Furthermore, GnRH improved estrus synchrony in beef cattle when injected 7 days prior to a GnRH-PGF_2*α*_ protocol [10], but failed to improve estrus response or synchrony of estrus in beef heifers when administered 7 days prior to a 14–19 day melengestrol acetate—PGF_2*α*_ treatment [11]. This difference in GnRH action may be associated with the development of persistent follicles during MGA feeding [2].

In studies where GnRH has been replaced by human Chorionic Gonadotropin (hCG) in Cosynch protocols, pregnancy rates have been greatly reduced in beef cows [12]. Controversially, other studies revealed significantly lower implantation rate and significantly higher rate of early pregnancy loss in patients induced to ovulate with a GnRH agonist when compared to those treated for the same purpose with hCG [13]. However, data reported by others [14] revealed no differences in the use of either GnRH or hCG to effectively induce ovulation in lactating dairy cows. Similar results were observed in studies where the second GnRH in TAI was replaced by hCG in dairy heifers [4]. In contrast, others reported [15] hCG being more effective than GnRH in its ability to ovulate follicles, increase volume of luteal tissue in the subsequent developing CL, and concentrations of progesterone in prepubertal heifers. 

It has been suggested that systemic levels of progesterone play a role in modulating the outcome in the use of hCG to induce ovulation in cattle [7]. Additionally, it is reported that expression of genes involved in the process of steroidogenesis was significantly increased in the CL of cows treated with pLH [16]. This seems to be in line with findings reported which revealed that serum levels of both estradiol and progesterone increase in response to an ovulatory dose of hCG in rats [17]. Moreover, high progesterone concentrations were observed in prepubertal Angus heifers [15] and in lactating dairy cows [18] in response to hCG-induced ovulations. 

Dynamics of follicular development and growth has been associated with LH in several research studies. Thus, an increase in follicular development has been observed in nonhuman primates after administration of exogenous LH [19]. Additionally, treatment of heifers with 3,000 IU of hCG on day 5 of the estrous cycle were reported to extend the life of the preovulatory follicles [20], to induce formation of a heavier accessory CL and to cause a linear increase of plasma progesterone from day 6 to 13 when compared to a GnRH agonist treatment [21]. Other researchers [22] stated that the role played by hCG is limited to the development of antral follicles. Nevertheless, it was also indicated that the need of LH for antral follicle maturation is dubious [19]. Furthermore, other investigators suggested that the response of the ovarian cells to hCG is mediated by the day of the estrous cycle when hCG is administered [23–26]. This trial was meant to evaluate the effects of hCG in advancing follicular development to ovulation in dairy heifers with progesterone levels similar to concentrations observed in early diestrus [27]. Other researchers observed alteration of the follicles dynamic in nonbred lactating cows receiving hCG on day 7 of the estrous cycle [28]; in that study, the time of emergence of the second wave of follicular growth was advanced in the group of cows receiving hCG treatment. Additionally, administration of hCG 7 days post insemination was suggested to improve embryo retention in cattle by boosting plasma progesterone concentration as a consequence of inducing formation of an accessory corpus luteum [29]. An additional objective of the present study was to elucidate the mechanism used by GnRH on ovarian tissue to maintain systemic progesterone levels despite the luteolytic effects of PGF_2*α*_ when both hormones are injected concurrently in cattle.

## 2. Materials and Methods

### 2.1. Experimental Design and Hormonal Protocol

Thirty-seven cycling postpubertal Holstein heifers were randomly allotted by weight, age, body condition score and after manual examination of the CL, to one of three treatments to test the hypothesis that injecting hCG after a simultaneous administration of GnRH and PGF_2*α*_ would advance follicular growth and ovulation time in heifers bearing a CL. Heifers in the study were selected from a larger pool of cycling animals by meeting the following criteria: (a) progesterone concentration in the blood of at least 3 ng/ml seven days before PGF_2*α*_ was administered (day-7); (b) heifers should be bearing a well-developed CL on day 0 (administration of PGF_2*α*_). The study was approved by the Institutional Animal Care and Use Committee of Alcorn State University (A3810-01). Body condition among heifers was established between 3 and 4 (BCS 1 = thin, BCS 5 = obese) prior to the beginning of the study. Heifers in the control group (PGF; *n* = 12) received a single injection of PGF_2*α*_ (25 mg im; Lutalyse; Pharmacia Upjohn Company) designed as day 0 of the study. The second group (PGF + GnRH; *n* = 13) additionally received an injection of GnRH (100 *μ*g im; Cystorelin; Sanofi Winthrop) immediately after the injection of PGF_2*α*_. Heifers in the third group (PGF + GnRH + hCG; *n* = 12) received the same GnRH and PGF_2*α*_ treatments as heifers in group 2; however, these heifers received an additional injection of hCG (1500 IU, im; Gonakor, Laboratorios Sanfer) 48 h after the injection of PGF_2*α*_ (day 2).

### 2.2. Collection of Blood Samples

Blood sample collection for progesterone and estrogen determination was initiated a week before the injection of PGF2*α* (day-7); subsequent samples were collected on days 0, 2, and 7. Blood samples were collected by tail vein puncture and were immediately centrifuged for plasma separation.

### 2.3. Progesterone and Estrogen Assays

Plasma samples were analyzed for progesterone concentration via radioimmunoassay. Progesterone assays were performed using a commercial enzyme immunoassay kit provided by Oxford Biomedical Research (Oxford, Michigan). This is an enzyme-linked immunosorbent assay that operates on the basis of competition of solid-phase RIA system relying upon competitive binding between a radioactive and nonradioactive antigen for a fixed number of antibody sites coated to the assay tubes. The specificity of this assay as reported by the manufacturer was 100% cross reactivity with progesterone, 2.5% with deoxycorticosterone, 2.0% with corticosterone, 2.0% with pregnenolone, 1.0% with androstenedione and less than 1.0% with other steroid hormones tested. The range of the progesterone assay used for this study was between 0 and 60 ng/ml. The assay displayed a sensitivity of 0.12 ng/ml and an average recovery rate of 97%. Average inter- and intra-assay coefficients of variability were 8.03 and 11.7 5%, respectively. 

Concentrations of estradiol in plasma samples were determined by radioimmunoassay. The procedure follows the basic principle of radioimmunoassay where there is competition between a radioactive and a nonradioactive antigen for a fixed number of antibody binding sites. The amount of [I-125]-labeled estradiol bound to the antibody is inversely proportional to the concentration of the estradiol present. Separation of free and bound antigen was achieved by using a double antibody system. Cross-reactivity of the estradiol antiserum has been detected at 2.40% with estrone, 3.40% with equilin, 2.56% with keto-estradiol and less than 1.0% with other steroid hormones tested.

### 2.4. Imaging of the Ovaries

Transrectal examination of follicular growth and day of ovulation was performed daily on both ovaries by real-time ultrasonography using an Aloka 500 v; it started on day 1 and continued up to day 10 of the study. Diameters of ovulating follicles were measured (mm) using a caliper on images obtained the day before ovulation; day of ovulation was determined by visual observation on images.

### 2.5. Statistical Analysis

Data collected on age, body weights, and body condition scores were analyzed using the analysis of variance (ANOVA) procedure. Data obtained on progesterone and estrogen levels in blood were analyzed using the GLM repeated measures analysis (SAS Institute., 1991). The correlation between hormone concentrations and days to ovulations was also evaluated using the SAS CORR procedure. Correlation between follicle size on days 0 and 2 with days to ovulations was also evaluated. LSD was used to test differences among and within treatments.

## 3. Results

There were not any significant differences (*P* > .05) in body weights (Kg; Mean ± S.E.) between heifers allocated in the PGF group (384.09 ± 18.4), PGF + GnRH group (387.27± 20.9) and the PGF + GnRH + hCG group (381.59 ± 13.6). Also, body condition scores for heifers were not different among the PGF (3.3 ± 0.1), PGF + GnRH (3.4 ± 0.1) and PGF + GnRH + hCG groups 3 (3.4 ± 0.1).

Table 1 shows means and standard errors for size (diameter, mm) of the ovulating follicle on days 0, 2 and on the day before ovulation as well as days to ovulation. Size of the ovulating follicles in the three experimental groups on days 0 and 2 were not different (*P* > .05). Additionally, size of the ovulating follicles of heifers in the PGF group 24 h before ovulation (11.0 ± 0.8 mm) not significantly different (*P* > .05) than the increases in size experienced by the ovulating follicles before rupture in PGF + GnRH (10.7 ± 1.1 mm) and PGF + GnRH + hCG groups (11.1 ± 0.6 mm). Days to ovulation were measured in heifers ovulating within the first 8 days of the study as the number of days between the injection of PGF_2*α*_ and the rupture of the ovulating follicle. The experimental group receiving hCG ovulated significantly earlier (3.64 ± 0.34 day; *P* < .05) than heifers allocated in the PGF (5.70 ± 0.42) and the PGF + GnRH (5.00 ± 0.56) groups. There were no significant differences in ovulating time between the PGF and PGF + GnRH heifers (*P* > .05). Size of follicles on days 0 and 2 in the ovulating heifers were not correlated to days to ovulation (*P* > .05).

Table 2 shows means and standard errors for progesterone concentrations between treatments and within treatments on heifers ovulating during the first 8 days of the study. Progesterone levels significantly dropped (*P* < .05) on day d2 (2.50 ± 0.77 ng/ml) in the PGF heifers in response to the injection of PGF_2*α*_ on day 0 (7.03 ± 0.96); then, it went up on day 7 (5.65 ± 0.83) but not significantly higher (*P* > .05). Progesterone also dropped significantly in PGF + GnRH heifers from 8.96 ± 1.48 on day 0 to 3.99 ± 1.0 on day 2 (*P* < .05). Levels observed on day 2 were not significantly different from those on day 7 (3.03 ± 0.71; *P* > .05) for that same group. On the other hand, PGF + GnRH + hCG heifers did not experience a significant decline in progesterone levels from day 0 (6.34 ± 1.29) to day 2 (3.78 ± 1.34; *P* > .05). Nevertheless, progesterone levels for this group of heifers on day 7 (9.04 ± 1.85), were significantly higher than levels observed on day 2 (*P* < .05). No significant differences (*P* > .05) were observed for plasma progesterone levels between the three experimental heifers on days-7, 0 and 2. Nevertheless, progesterone levels were significantly higher on day 7 for heifers in the PGF + GnRH + hCG group compared to the PGF + GnRH group (*P* < .05) on that day. The increases in progesterone concentrations (ng/ml) experienced by the PGF (3.16 ± 1.03) and the PGF + GnRH + hCG (5.26 ± 1.33) groups from day 2 to day 7, were not significantly different (*P* > .5); however, both groups had a significant higher increase in progesterone when compared to the change experienced on those same days by heifers receiving PGF + GnRH (−0.96 ± 1.07 ng/ml). 

Table 3 shows number of heifers ovulating by treatment and by day of the study. Ovulation occurred in 7 out of the 11 ovulating heifers in the PGF group between day 5 and 6 while the majority (8/11) of heifers in the PGF + GnRH + hCG group ovulated between day 3 and 4. However, the highest number of ovulations in the PGF + GnRH animals occurred on day 5 of the experiment (5/11). Other animals receiving this experimental treatment ovulated more than 48 h apart from the animals ovulating on day 5. Out of the total number of heifers alloted to each experimental group, one heifer in the PGF and one in PGF + GnRH + hCG group did not ovulate within the first 8 days of the study; two also did not ovulate in PGF + GnRH group within the same period.

Estrogen concentrations in plasma were not statistically different (*P* > .05) between heifers in the three experimental groups on days-7, 0, 2, and 7. However, overall estrogen concentrations and concentrations on days 2 and 7 were correlated (*P* < .05) with days to ovulation (Table 4). Table 4 also shows a correlation between overall and day 7 plasma progesterone with days to ovulation (*P* < .05).

## 4. Discussion

There were no significant differences in size of the ovulating follicles on days 0, 2 or on the day before ovulation amongst heifers allotted to the three experimental treatments. Nevertheless, follicles in heifers receiving hCG, reached the ovulating size earlier than the other two groups of heifers in the study; as a consequence, these heifers also ovulated significantly earlier than animals in the other two experimental groups. This data suggests that administration of hCG plays a role in advancing follicular growth to ovulation and in promoting follicular growth. Levels of progesterone in this group were 3.78 ± 1.34 ng/ml on day 2 when hCG was administered and went up 5 days later (day 7) to 9.04 ± 1.85. However, data previously reported revealed that progesterone concentrations of 1.20 ng/ml inhibited ovulation in lactating cows treated 12 days apart with two injections of PGF_2*α*_ and an injection of estradiol cypionate two days after the first injection of PGF_2*α*_ [7]. These investigators concluded that progesterone reduced the ability of endogenous or exogenous estradiol to induce a preovulatory LH surge and ovulation. Furthermore, other investigators reported that cows with progesterone concentrations greater than 1 ng/ml before treatment with either hCG or GnRH tend to ovulate less often than those having progesterone levels below 1 ng/ml [30]. Nevertheless, the group receiving the PGF + GnRH + hCG treatment in the present study ovulated earlier than the other two experimental groups even though progesterone levels were higher (3.78 ± 1.34) than 1 ng/ml. Thus, our findings demonstrate that the mechanism previously proposed [7] can be overridden by exogenous administration of hCG by acting directly on follicular tissue [31]. This data also indicate that cows receiving GnRH, followed 48 h later with hCG, would advance to ovulation at progesterone concentrations much higher than 1 ng/ml at time of the hCG treatment. 

Our results agree with data reported by researchers [17] who induced ovulation in women, beef cows [30], and dairy heifers [32] treated with hCG injections. In addition, our data is also in line with research findings which suggest that hCG selectively stimulates folliculogenesis and induces ovulation in women [33]. Furthermore, other investigators [22] concluded that hCG actions are more selectively associated with follicles in advanced stages of development. 

The criteria previously described for selecting heifers participating in this study resulted in progesterone concentrations for the three groups on day 0 comparable to those observed in heifers with a functional CL (8.2 ± 0.3 ng/ml) at the end of a 14-day treatment with melengestrol acetate [27, 34]. Mean progesterone concentrations observed in this study for the PGF, PGF + GnRH, and PGF + GnRH + hCG heifers (7.03 ± 0.96; 8.96 ± 1.48; 6.34 ± 1.29 ng/ml) on day 0 are higher than those reported in a earlier study [27] for heifers in early diestrus (between 1 and <3 ng/ml). Thus, this indicates heifers involved in the present trial were beyond the early stage of diestrus on day 0 of this study. 

As expected, progesterone levels dropped in animals in all three experimental groups in response to the injection of PGF_2*α*_. Nevertheless, a higher decrease in progesterone was expected in the PGF group 48 h after the injection. While some heifers had progesterone concentrations at 48 h after PGF_2*α*_ immediately above the sensitivity level of the assay, some heifers in this group had levels higher than 1 ng/ml. This may be due to an incomplete luteolysis at the time the blood sample was collected influenced by the level of progesterone and by the time of the day when PGF_2*α*_ was administered. A previous study [27] has suggested a 72 h period for complete regression of a functional CL after a single injection of PGF_2*α*_ which is affected by concentrations of progesterone. 

The decrease in progesterone experienced on day 2 by heifers receiving GnRH in response to PGF_2*α*_ is in agreement with previous data reported by our laboratory. In that study [6], it was clearly demonstrated that GnRH prevents total luteolysis in cattle when injected right immediately after PGF_2*α*_ and consequently maintaining high levels of progesterone for a period of 10 days. Furthermore, images obtained in the present study also revealed the anti-luteolytic actions of GnRH when concurrently injected with PGF_2*α*_.

Heifers in the PGF + GnRH + hCG group were the only group experiencing a significant increase in plasma progesterone on day 7 after the decline of this hormone in response to PGF_2*α*_. This was the case when considering all heifers in the group or for just those ovulating within the first 8 days of the trial. Additionally, these animals experienced an earlier ovulation compared to the other two experimental groups; consequently, this group had an older corpus luteum at day 7 which was active enough to establish the difference observed in progesterone concentrations. On the other hand, plasma progesterone concentrations observed in heifers in the PGF + GnRH group remained steady after the PGF_2*α*_ and GnRH treatments. This may be attributed to the effects of GnRH previously observed by our laboratory. We reported that GnRH impairs luteolysis in dairy heifers when injected concurrently with PGF_2*α*_ [6]. 

No significant differences were detected on days 0 and 2 (*P* > .00) in progesterone concentrations between the three groups. Heifers in the PGF + GnRH + hCG group had significant higher levels of this hormone than the PGF + GnRH animals on day 7. On the other hand, there was only a trend toward higher progesterone levels in heifers receiving hCG when compared to PGF animals on that same day of this study. The difference observed in progesterone levels on day 7 between the two groups receiving GnRH was mainly the result of an early ovulation and consequently due to the presence of a more mature luteal tissue; however, the restraining effects on progesterone secretion exerted by the exogenous GnRH on luteal tissue in the PGF + GnRH group also contributed to this difference. These GnRH haltering effects were not present in the group of heifers receiving only PGF; consequently, it resulted in a more active progesterone secretion by the luteal tissues of these animals. Therefore, the difference in progesterone levels on day 7 between the PGF and the PGF + GnRH + hCG was smaller and consequently not significantly different. 

 Estrogen concentrations were not affected by any of the three experimental treatments (*P* > .05). However, other investigators [35] have observed an increase in estrogen after luteolysis in cattle which could have been reflected in the PGF animals of this study.

## 5. Conclusion

This data suggests that treatment of PGF_2*α*_ concurrently with GnRH followed 48 h later with hCG further accelerates follicular growth, advancing follicular growth to ovulation. This can be evidenced by the sharp increase in progesterone observed after hCG (day 7) as the result of an early ovulation and consequently due to the presence of a more mature luteal tissue. Authors of this paper additionally concluded that hCG induces ovulation at progesterone levels higher than those previously reported to prevent it [7]. Furthermore, these research findings indicate that systemic progesterone does not seem to impair the ability of hCG to induce ovulation in cattle. This data also suggests a potential application of hCG in hormonal protocols designed to time ovulation in cattle.

## Figures and Tables

**Table 1:** Means and standard errors for size (mm) of ovulating follicle and days to ovulation.

Treatment	*N*	Follicle size			Days to ovulation*
		Day 0	Day 2	Preovulation**	
PGF	11	2.79 ± 0.99^a^	2.80 ± 0.09^a^	11.0 ± 0.8^a^	5.70 ± 0.42^a^
PGF + GnRH	11	2.78 ± 1.04^a^	2.82 ± 0.08^a^	10.7 ± 1.1^a^	5.00 ± 0.56^a^
PGF + GnRH + hCG	11	2.77 ± 1.04^a^	2.81 ± 0.09^a^	11.1 ± 0.6^a^	3.64.1 ± 0.34^b^

^a^Means within the same column lacking a common superscript are significantly different (*P* < .05)

*Days from injection of PGF_2*α*_

**24 h before ovulation.

**Table 2:** Means and standards errors for progesterone concentrations in ovulating heifers by treatment and by day (ng/ml).

Treatment	*N*	Day-7	Day 0*	Day 2	Day 7
PGF	11	7.19 ± 1.68^aɛ′^	7.03 ± 0.96^aɛ′^	2.50 ± 0.77^a*£*^	5.65 ± 0.83^abɛ′*£*^
PGF + GnRH	11	6.37 ± 1.17^aɛ′*£*^	8.96 ± 1.48^aɛ′^	3.99 ± 1.00^a*£γ*^	3.03 ± 0.71^a*γ*^
PGF + GnRH + hCG	11	5.72 ± 1.18^aɛ′*£*^	6.34 ± 1.29^aɛ′*£*^	3.78 ± 1.34^a*£*^	9.04 ± 1.85^bɛ′^

^a,b^Means within the same column lacking a common superscript differ (*P* < .05)

^ɛ′,*£*,*γ*^Means within the same row lacking a common symbol differ (*P* < .05)

*N*: Number of heifers

*Injection of PGF_2*α*_.

**Table 3:** Number ovulations by treatment and by day.

Treatment	*N*	Day 1	Day 2	Day 3	Day 4	Day 5	Day 6	Day 7	Day 8
PGF	11	0	0	1	0	3	4	1	2
PGF + GnRH	11	1	1	2	0	5	0	2	0
PGF + GnRH + hCG	11	1	0	5	3	1	1	0	0

**Table 4:** Pearson correlation coefficients: days to ovulation X hormone concentration by day (correlation value/*P*-value).

Hormone	Day-7	Day 0	Day 2	Day 7	Overall
Progesterone	−0.02975	−0.10803	−0.33603	−0.39587	−0.25876
(ng/ml)	0.1040	0.5629	0.0646	0.0275	0.0037
Estrogen	−0.05859	0.23097	0.41938	0.37511	0.26333
(pg/ml)	0.7542	0.2113	0.0188	0.0376	0.0275

Significant Different at *P* < .05.

## References

[1] Lamb GC, Stevenson JS, Kesler DJ, Garverick HA, Brown DR, Salfen BE (2001). Inclusion of an intravaginal progesterone insert plus GnRH and prostaglandin F2*α* for ovulation control in postpartum suckled beef cows. *Journal of Animal Science*.

[2] Sá Filho OG, Patterson DJ, Vasconcelos JL (2009). Development of estrous synchronization protocols using melengestrol acetate in *Bos indicus* cattle. *Journal of Animal Science*.

[3] Rose C, Fuquay J, Moore A (2000). Evaluation of injection intervals in a modified ovulation-synchronization protocol in dairy heifers. *Journal of Animal Science*.

[4] Schmitt ÉJ-P, Diaz T, Drost M, Thatcher WW (1996). Use of gonadotropin-releasing hormone agonist or human chorionic gonadotropin for timed insemination in cattle. *Journal of Animal Science*.

[5] Martins JPN, Policelli R, Pursley JR (2009). Dynamics of luteolysis using two PGF_2*α*_ analogs and subsequent differences in fertility. *Journal of Animal Science*.

[6] Harper R, Bennett WA,  Cuadra EJ, Vaughn CF, Watson CE, Whitworth NS (2008). Effects of GnRH in combination with PGF2*α* on the dynamic of follicular and luteal endocrine cells in post-pubertal Holstein heifers. *Journal of Livestock Science*.

[7] Hatler TB, Hayes SH, Ray DL, Reames PS, Silvia WJ (2008). Effect of subluteal concentrations of progesterone on luteinizing hormone and ovulation in lactating dairy cows. *Veterinary Journal*.

[8] Twagiramungu H, Guilbault LA, Proulx J, Ramkumar R, Dufour JJ (1994). Histological populations and atresia of ovarian follicles in postpartum cattle treated with an agonist of gonadotropin-releasing hormone. *Journal of Animal Science*.

[9] Schmitt ÉJ-P, Diaz T, Barros CM (1996). Differential response of the luteal phase and fertility in cattle following ovulation of the first-wave follicle with human chorionic gonadotropin or an agonist of gonadotropin-releasing hormone. *Journal of Animal Science*.

[10] Kojima FN, Wood SL,  Smith MF, Patterson DJ (2000). Does pretreatment with GnRH prior to a GnRH- PGF_2*α*_ (PG) improve synchronization of estrus in beef cattle?. *Journal of Animal Science*.

[11] Wood SL, Lucy MC, Smith MF (2000). Estrus and fertility in beef heifers synchronized with melengestrol acetate (MGA) and prostaglandin F_2*α*_ (PG) with or without GnRH. *Journal of Animal Science*.

[12] Geary TW, Salverson RR, Whittier JC (2001). Synchronization of ovulation using GnRH or hCG with the CO-Synch protocol in suckled beef cows. *Journal of Animal Science*.

[13] Humaidan P, Bredkjær HE, Bungum L (2005). GnRH agonist (buserelin) or hCG for ovulation induction in GnRH antagonist IVF/ICSI cycles: a prospective randomized study. *Human Reproduction*.

[14] Stevenson JS, Tenhouse DE, Portaluppi MA, Lloyd A (2000). Post-AI intervention in lactating dairy cattle. I. Ovarian responses to GnRH, hCG, and exogenous progesterone (CIDR). *Journal of Animal Science*.

[15] Dahlen CR, Larson JE, Marquezini G, Lamb JC (2007). Effects of human chorionic gonadotropin (hCG) and gonadotropin releasing hormone (GnRH) on follicle and corpus luteum dynamics and concentrations of progesterone in pre-pubertal Angus heifers. *Journal of Animal Science*.

[16] Ponce-Barajas P, Colazo MG, Kastelic JP, Dyck MK, Ambrose DJ (2007). Real time PCR quantification of mRNA expression in the corpus luteum of cows induced to ovulate following different hormonal treatments. *Journal of Animal Science *.

[17] Roby KF (2001). Alterations in follicle development, steroidogenesis, and gonadotropin receptor binding in a model of ovulatory blockade. *Endocrinology*.

[18] De Rensis F, Valentini R, Gorrieri F, Bottarelli E, Lopez-Gatius F (2008). Inducing ovulation with hCG improves the fertility of dairy cows during the warm season. *Theriogenology*.

[19] Stouffer RL, Zelinski-Wooten MB (2004). Overriding follicle selection in controlled ovarian stimulation protocols: quality vs quantity. *Reproductive Biology and Endocrinology*.

[20] Diaz T, Schmitt EJ-P, De La Sota RL, Thatcher M-J, Thatcher WW (1998). Human Chorionic Gonadotropin-induced alterations in ovarian follicular dynamics during the estrous cycle of heifers. *Journal of Animal Science*.

[21] Schmitt ÉJ-P, Barros CM, Fields PA (1996). A cellular and endocrine characterization of the original and induced corpus luteum after administration of a gonadotropin-releasing hormone agonist or human chorionic gonadotropin on day five of the estrous cycle. *Journal of Animal Science*.

[22] Wang Y, Newton H, Spaliviero JA (2005). Gonadotropin control of inhibin secretion and the relationship to follicle type and number in the *hpg* mouse. *Biology of Reproduction*.

[23] Seguin BE, Oxender WD, Britt JH (1977). Effect of human chorionic gonadotropin and gonadotropin releasing hormone on corpus luteum function and estrous cycle duration in dairy heifers. *American Journal of Veterinary Research*.

[24] Breuel KF, Spitzer JC, Henricks DM (1989). Systemic progesterone concentration following human chorionic gonadotropin administration at various times during the estrous cycle in beef heifers. *Journal of Animal Science*.

[25] Howard HJ, Scott RG, Britt JH (1990). Associations among progesterone, estradiol-17*β*, oxytocin and prostaglandin in cattle treated with hCG during diestrus to extend corpus luteum function. *Prostaglandins*.

[26] Fricke PM, Reynolds LP, Redmer DA (1993). Effect of human chorionic gonadotropin administered early in the estrous cycle on ovulation and subsequent luteal function in cows. *Journal of Animal Science*.

[27] Bridges GA, Portillo GE, de Araujo JW, Thatcher WW, Yelich JV (2005). Efficacy of either a single or split treatment of PGF2*α* after a 14 day melengestrol acetate treatment to synchronized estrus and induce luteolysis in *Bos indicus* X Bos taurus heifers. *Theriogenology*.

[28] Sianangama PC, Rajamahendran R (1996). Effect of hCG administration on day 7 of the estrous cycle on follicular dynamcs and cycle length in cows. *Theriogenology*.

[29] Rajamahendran R, Sianangama PC (1992). Effect of human chorionic gonadotrophin on dominant follicles in cows: formation of accessory corpora lutea, progesterone production and pregnancy rates. *Journal of Reproduction and Fertility*.

[30] Burns MG, Buttrey BS, Dobbins CA (2008). Evaluation of human chorionic gonadotropin as a replacement for gonadotropin-releasing hormone in ovulation-synchronization protocols before fixed timed artificial insemination in beef cattle. *Journal of Animal Science*.

[31] De Rensis F, López-Gatius F, García-Ispierto I, Techakumpu M (2010). Clinical use of human chorionic gonadotropin in dairy cows: an update. *Theriogenology*.

[32] Howard HJ, Britt JH (1990). Prostaglandin F-2*α* causes regression of an hCG-induced corpus luteum before day 5 of its lifespan in cattle. *Journal of Reproduction and Fertility*.

[33] Filicori M, Cognigni GE, Tabarelli C (2002). Stimulation and growth of antral ovarian follicles by selective LH activity administration in women. *Journal of Clinical Endocrinology and Metabolism*.

[34] Funston RN, Olson JL, Lipsey RJ, Geary TW, Roberts AJ (2003). Effect of hCG administration approximately 5 d after artificial insemination on progesterone concentrations and AI conception rates in beef heifers. *Journal of Animal Science*.

[35] Echternkamp SE, Roberts AJ, Lunstra DD, Wise T, Spicer LJ (2004). Ovarian follicular development in cattle selected for twin ovulations and births. *Journal of Animal Science*.

